# Induced Mutagenesis Improves Grain Protein and Micronutrient (Fe and Zn) Content in Spring Wheat (*Triticum aestivum* L.)

**DOI:** 10.3390/biology15110891

**Published:** 2026-06-04

**Authors:** Doktyrbay Gulina, Kenzhebayeva Saule, Zharassova Dinara, Atabayeva Saule, Abdulzhanova Malika, Shoinbekova Sabina, Asrandina Saltanat, Yevloyeva Khava

**Affiliations:** 1 Department of Biotechnology, Faculty of Biology and Biotechnology, Al-Farabi Kazakh National University, Almaty 050040, Kazakhstan; kenzhebss@gmail.com (K.S.); saule.atabayeva@kaznu.edu.kz (A.S.); malika.abdulzhanova@kaznu.edu.kz (A.M.); sabina.shoinbekova@kaznu.edu.kz (S.S.); saltanat.asrandina@kaznu.edu.kz (A.S.); 2 Mangyshlak Experimental Botanical Garden, Aktau 130000, Kazakhstan; d.zharass@gmail.com; 3 Department of Chemistry and Biotechnology, Shokan Ualikhanov Kokshetau University, Kokshetau 020000, Kazakhstan; kyevloyeva@shokan.edu.kz

**Keywords:** gamma irradiation, wheat biofortification, iron (Fe), zinc (Zn), phytic acid, grain protein content, induced mutagenesis

## Abstract

Improving grain nutritional quality without reducing yield remains a major challenge in wheat breeding. This study aimed to identify advanced mutant lines of spring wheat with enhanced grain protein, iron (Fe), and zinc (Zn) contents combined with reduced phytate levels to improve mineral bioavailability. Mutant lines were developed from the spring wheat cultivar Zhenis using gamma irradiation (100 and 200 Gry) and evaluated for yield-related traits, grain morphometry, and nutritional parameters. Significant phenotypic and genetic variation was observed among the M_5_ mutant lines. Grain protein content ranged from 13.23% to 15.63%, and 46.7% of the lines showed significantly higher protein levels than the parent cultivar. Likewise, 43.3% of the mutant lines showed increases in grain iron and zinc contents of up to 3.4- and 2.94-fold, respectively, compared to the control. Phytate-to-mineral molar ratios were significantly reduced, indicating improved mineral bioavailability. Correlation analysis revealed positive associations between micronutrient accumulation and grain morphometric traits, particularly grain area. No strong negative relationship between nutritional quality and yield-related traits was detected in the selected lines. These results demonstrate that gamma-induced mutagenesis is an effective approach for developing biofortified wheat genotypes with improved nutritional quality and stable agronomic performance.

## Simple Summary

Micronutrient deficiency, especially iron and zinc deficiency, remains a major global nutritional problem. Wheat is one of the most important staple crops worldwide and can be improved to provide higher nutritional value. In this study, mutant lines of spring wheat developed by gamma irradiation were evaluated for grain protein, iron, zinc, and phytate content. Several mutant lines showed increased protein and micronutrient content together with reduced phytate levels, which may improve mineral bioavailability. Importantly, these nutritional improvements were achieved without major negative effects on yield-related traits. The identified mutant lines may serve as valuable genetic resources for wheat biofortification programs aimed at developing more nutritious wheat cultivars and improving human health.

## 1. Introduction

Bread wheat (*Triticum aestivum* L.) is one of the most important staple crops worldwide and a major source of calories and protein for human populations. However, imbalanced dietary intake and limited micronutrient availability have led to widespread deficiencies, resulting in serious health problems, including impaired growth, weakened immune function, and increased socioeconomic burden [1,2,3]. Therefore, improving the nutritional quality of staple crops such as wheat is a key strategy for addressing global malnutrition and enhancing human health.

Micronutrient deficiencies, particularly of iron (Fe) and zinc (Zn), remain a major global health concern, affecting billions of people and contributing to anemia, impaired cognitive development, and reduced immunity [4,5]. These deficiencies are especially prevalent in regions where cereal-based diets dominate and mineral bioavailability is low. Although wheat is a primary staple crop, it typically contains insufficient levels of bioavailable Fe and Zn. In contrast, wild relatives of wheat, such as *Triticum turgidum* ssp. dicoccoides, represent valuable genetic resources with significantly higher micronutrient content [6,7]. However, modern cultivars often exhibit reduced micronutrient density due to intensive selection for yield, highlighting the need for effective strategies to enhance grain nutritional quality.

Conventional breeding programs have primarily focused on improving yield and disease resistance, often resulting in reduced genetic diversity and limited progress in enhancing nutritional traits [8,9]. Moreover, negative correlations between yield and grain nutritional traits, particularly grain protein content (GPC), have further complicated breeding efforts [10,11,12]. This narrow genetic base restricts the potential for developing varieties that combine high yield with improved nutritional quality.

In this context, induced mutagenesis has emerged as a powerful tool for generating novel genetic variation. Gamma irradiation, in particular, has been widely used to create beneficial mutations that enhance agronomic performance, stress tolerance, and grain quality without significantly altering the genetic background of elite cultivars [13,14]. Importantly, mutagenesis enables the simultaneous modification of multiple complex traits, including micronutrient accumulation and grain composition, which are difficult to improve through conventional breeding alone.

Grain protein content (GPC) is a key determinant of wheat nutritional and technological quality, strongly influencing end-use performance. However, improving GPC remains challenging due to its complex genetic control and its frequently reported negative association with yield. Similarly, grain size and shape are important yield-related traits that influence kernel weight, milling efficiency, and overall productivity [15,16]. Optimizing these traits while maintaining or enhancing nutritional quality remains a major challenge due to complex genetic interactions.

In addition to low micronutrient content, the presence of antinutritional compounds such as phytic acid (PA) further limits mineral bioavailability in wheat grain. Phytate strongly chelates Fe and Zn, reducing their absorption in the human digestive system [17,18]. Therefore, reducing phytate content while increasing mineral concentration is a critical objective in biofortification strategies.

Despite significant progress in wheat biofortification, most studies have focused on individual traits, such as micronutrient concentration or phytate reduction, rather than their combined improvement. The simultaneous enhancement of Fe and Zn contents, reduction of phytic acid, and maintenance of yield-related and morphometric traits remains insufficiently explored, particularly in mutant wheat populations [19].

Therefore, the aim of this study was to identify advanced wheat mutant lines combining increased grain protein, enhanced iron and zinc accumulation, and reduced phytate levels.

## 2. Materials and Methods

### 2.1. Plant Material and Application of Induced Mutagenesis

Seeds of the spring bread wheat variety Zhenis (*Triticum aestivum* L.) were irradiated with doses of 100 and 200 Gry using a ^60^Co source at the Kazakh Nuclear Centre. The seeds were sown immediately after irradiation to obtain M_2_ plants. The M_1_ generation was cultivated in the experimental fields of the Kazakh Research Institute of Agriculture and Crop Production located near Almaty, Kazakhstan (43°15′ N, 76°54′ E; 550 m above sea level). The experiments were carried out under typical continental climatic conditions of southeastern Kazakhstan. The soil in the experimental area was characterized as moderately fertile loamy soil. Standard agronomic management practices commonly used for spring wheat cultivation in the region were followed throughout the study. Mineral fertilizers were applied prior to sowing in accordance with local agricultural guidelines, while irrigation and weed management were performed when necessary during plant growth.

Single spikes were harvested from each plant to develop the M_2_ generation, and subsequent selection of superior lines from M_1_ to M_5_ generations was carried out based on individual plant performance. The M_3_ and M_4_ generations were grown in randomized block designs with three replications. Selected lines were evaluated in comparison with the parent variety to identify superior mutants.

Selection criteria included grain weight per spike (GWS) and grain weight per plant (GWP), assessed in the M_3_ and M_4_ generations (2011–2012), based on the values of the parent cultivar Zhenis grown under identical conditions. In 2011, the parent line exhibited mean values of GWS = 1.26 ± 0.51 g and GWP = 1.85 ± 0.61 g. For the M_4_ generation, threshold values for selection were set at GWS ≥ 1.4 g and GWP ≥ 2.3 g. Seeds from selected mutants were advanced individually across generations. After harvesting the M_5_ plants, 15 mutant lines derived from the 100 Gry treatment were selected: 5(4), 6(4), 6(5), 6(13), 13(3), 18(5), 24(1), 24(2), 25(2), 26(6), 26(7), 26(9), 26(10), 30(1), and 36(1). Additionally, 15 mutant lines derived from the 200 Gry treatment were selected: 43(1), 43(3), 43(4), 45(1), 45(2), 45(3), 48(3), 49(2), 49(4), 49(6), 50(7), 51(1), 51(2), 51(8), and 53(2). All lines were subjected to field trials alongside the parent cultivar Zhenis under identical conditions. Each line was grown in three replicates with standard plot dimensions and planting density.

Yield-related traits, including GWP, grain number per spike (GNS), and GWS, were recorded. Thousand-grain weight (TGW) was calculated as the mean weight of three sets of 100 grains per line.

### 2.2. Grain Morphometric Analysis

Morphometric analysis was performed using the WinRHIZO image analysis system (version 1.38, Regent Instruments Inc., Québec, QC, Canada). Grain length (GL), grain width (GW), and grain area (GA) were measured using 50–60 grains per line. The GL/GW ratio was also calculated.

### 2.3. Estimation of Grain Protein Content

Grain protein content was determined using near-infrared reflectance (NIR) spectroscopy (ZX50 Portable Grain Analyzer, Hagerstown, MD, USA) with appropriate calibration software. Measurements were conducted on whole grains, with three replicates of 25 grains per line.

### 2.4. Evaluation of Iron and Zinc Content

Grain samples from M_5_ mutant lines (100 and 200 Gry treatments) and the parent cultivar Zhenis were washed with 0.1% sodium dodecyl sulfate and rinsed multiple times with deionized water. Samples were dried to constant weight at 65–70 °C and ground using a mixer mill (Retsch MM400, Hamburg, Germany). Approximately 0.2 g of each sample was digested using a mixture of nitric acid (65%) and hydrogen peroxide (30%) (5:1, *v*/*v*) in a digestion system (K-438 digestion unit and K-415 scrubber, Büchi Labortechnik AG, Flawil, Switzerland). Digestion was performed using a temperature program: 70 °C for 40 min, 90 °C for 45 min, 130 °C for 4 h, and 150 °C for 1 h. After cooling, samples were diluted to 20 mL with double-distilled water. Iron and zinc contents were determined using flame atomic absorption spectroscopy (Analytik Jena NovaAA350, Jena, Germany). Accuracy was verified using certified reference materials. Three independent replicates were analyzed.

### 2.5. Determination of Phytic Acid Content in Grain

Phytic acid (PA) content was determined using a modified Megazyme enzymatic assay kit protocol (Megazyme K-PHYT 05/17, 2017, Bray, Ireland). A 2.0 g grain sample was ground using a mixer mill (Retsch MM400, Hamburg, Germany). The obtained flour was transferred into a 15 mL Falcon tube, and 10 mL of 0.66 M HCl was added. The samples were incubated on a shaker at room temperature for 8 h. Subsequently, 1 mL of the extract was centrifuged at 13,000 rpm for 8 min using an Eppendorf Centrifuge 5417R (Hamburg, Germany). Then, 0.3 mL of the supernatant was transferred into a new Eppendorf tube and mixed with NaOH (0.75 M) and HCl (0.66 M) solution at a 2:1 ratio. For the control sample, 0.1 mL of 0.66 M HCl was added. Absorbance was measured at 655 nm using an Eppendorf BioPhotometer Plus (Hamburg, Germany), and phytic acid content was calculated according to the Megazyme protocol. The phytic acid concentration was expressed as mg kg^−1^.

### 2.6. Calculation of PA:Fe and PA:Zn Molar Ratios

The molar content of phytic acid (PA), Fe, and Zn were calculated by dividing their respective content by their molecular or atomic weights (660.04 g mol^−1^ for PA, 55.85 g mol^−1^ for Fe, and 65.4 g mol^−1^ for Zn). Subsequently, the PA:Fe and PA:Zn molar ratios were calculated.

### 2.7. Statistical Analysis

Statistical analyses were performed using GenStat software (10th edition). Analysis of variance (ANOVA) was used to evaluate the significance of differences among the studied wheat lines. Prior to ANOVA, the assumptions of normality and homogeneity of variance were assessed using the Shapiro–Wilk and Levene’s tests, respectively. Dunnett’s test was subsequently applied for multiple comparisons between mutant lines and the parental cultivar Zhenis. Correlation analysis between grain productivity and quality-related parameters were also conducted. All measurements were performed using three biological replicates, and results are presented as mean ± standard deviation (SD). Statistical significance was determined at *p* < 0.05, *p* < 0.01, *p* < 0.001.

## 3. Results

### 3.1. Yield-Related Traits

Mutant lines exhibited substantial variation in yield-related traits (Table 1). Grain weight per plant (GWP) ranged from 1.64 to 14.74 g, with a mean value of 4.7 ± 1.4 g (*n* = 30). In particular, lines 5(4), 6(4), 45(1), and 51(8) showed significantly higher GWP values (3.1–3.9-fold) compared with the parental cultivar Zhenis, indicating their strong potential for improved yield performance. In contrast, grain number per spike (GNS) and grain weight per spike (GWS) showed no significant differences between mutant lines and the parent, with values ranging from 27.5 to 54.1 and 1.0 to 2.25 g, respectively. The thousand-grain weight (TGW) ranged from 34.92 to 74.65 g (mean 42.88 ± 7.37 g), and mutant lines 5(4), 24(2), 43(4), 49(4), and 49(6) demonstrated significantly higher values (1.4–2-fold), indicating strong potential for yield improvement.

### 3.2. Grain Morphometric Traits

Grain morphometric traits showed substantial variation among mutant lines (Table 2). GA ranged from 18.9 to 23.0 mm^2^, with a mean of 21.11 ± 1.52 mm^2^, and was significantly higher than in the parental cultivar Zhenis, with increases of up to 31.9%. Several mutant lines (56.7%) exhibited enlarged grain dimensions, including GL and GW, indicating their potential contribution to improved yield performance.

The observed variation reflects irradiation-induced phenotypic diversity, particularly for grain size traits. Notably, several lines exceeded the parent cultivar by approximately 1.3-fold in GA. Correlation analysis further revealed weak to moderate positive associations between grain morphometric parameters and grain quality traits, including GPC, Fe, and Zn contents, with correlation coefficients ranging from 0.17 to 0.24, suggesting a potential genetic linkage between grain size and nutrient accumulation (Figure 3 and Table 4).

### 3.3. Grain Micronutrient Accumulation and Relationships

**Figure 1:**
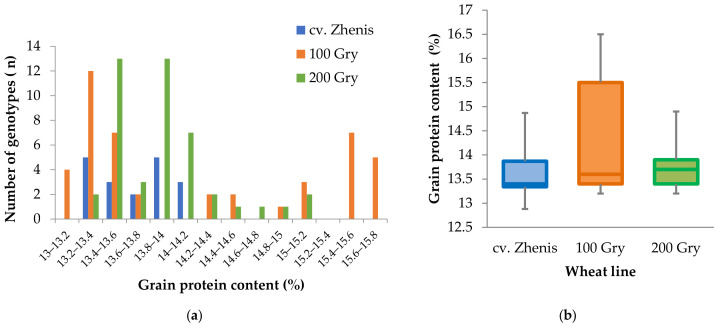
Effect of γ-irradiation (100 and 200 Gry) on the distribution and variability of grain protein content (%) in wheat lines. (**a**) Histogram of genotype frequency; (**b**) Box plot of protein content variation.

Grain protein content varied among the studied wheat lines, ranging from approximately 13.1% to 15.8% (Figure 1a). In particular, mutant lines 5(4), 24(1) and 26(9) showed the highest protein content relative to the parental cultivar. Mutant lines treated with 100 Gry and 200 Gry irradiation showed broader variability compared with the parental cultivar cv. Zhenis. Boxplot analysis (Figure 1b) illustrated variation in protein content distribution among the analyzed lines.

**Figure 2:**
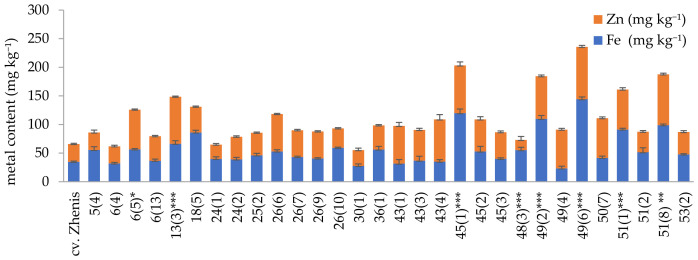
Grain iron (Fe) and zinc (Zn) content (mg kg^−1^) in cv. Zhenis and M_5_ mutant lines generated by 100 and 200 Gry gamma irradiation. Bars represent mean ± SD. Asterisks indicate significant differences compared to the control (* *p* < 0.05, ** *p* < 0.01, *** *p* < 0.001).

Substantial variation in grain iron (Fe) and zinc (Zn) content was observed among the mutant lines relative to the parental cultivar cv. Zhenis (Figure 2). Grain Fe concentration ranged from approximately 23.29 to 144.27 mg kg^−1^, while Zn concentration varied between 17.90 and 91.40 mg kg^−1^, indicating broad irradiation-induced variability.

As shown in Figure 2, a considerable proportion of mutant lines exhibited significantly higher micronutrient content relative to the parental cultivar (*p* ≤ 0.05). In particular, mutant lines 45(1), 49(6), and 51(8) demonstrated the highest Fe and Zn accumulation, with up to 3.4-fold increases in Fe and nearly 3-fold increases in Zn, confirming the effectiveness of induced mutagenesis for enhancing mineral accumulation.

**Figure 3:**
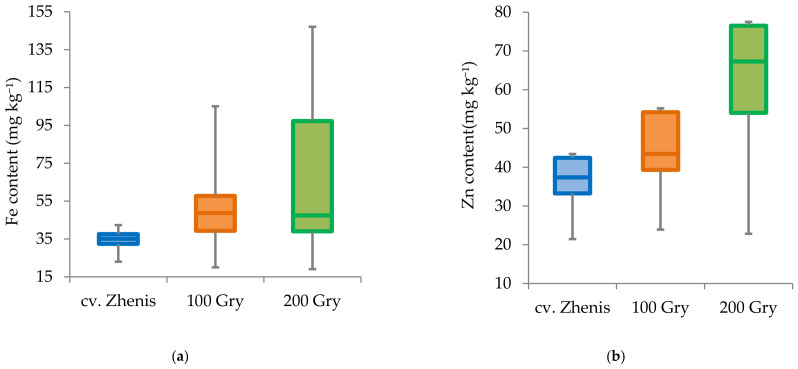
Effects of gamma irradiation (100 and 200 Gry) on grain Fe (**a**) and Zn (**b**) content (mg kg^−1^) in cv. Zhenis and M_5_ mutant lines. Boxplots represent median values and interquartile ranges.

Boxplot analysis (Figure 3) illustrated variation in distribution patterns among the studied wheat lines. The parental cultivar displayed narrow ranges with lower median values (Fe ≈ 30–35 mg kg^−1^; Zn ≈ 30–40 mg kg^−1^), whereas mutant populations showed broader variability. The 200 Gry treatment exhibited the widest range and highest upper values (Fe > 130 mg kg^−1^; Zn > 70 mg kg^−1^), while the 100 Gry treatment showed moderate increases with more stable distributions.

Correlation analysis (Table 4) demonstrated a positive relationship between Zn and Fe content, with increasing strength at higher irradiation doses (R^2^ = 0.17, 0.20, and 0.24 for control, 100 Gry, and 200 Gry, respectively). This suggests partial co-regulation of micronutrient accumulation. Overall, induced mutagenesis enhanced both the magnitude and coordination of Fe and Zn accumulation, highlighting its potential for wheat biofortification.

### 3.4. Phytate Content and Mineral Bioavailability

**Figure 4:**
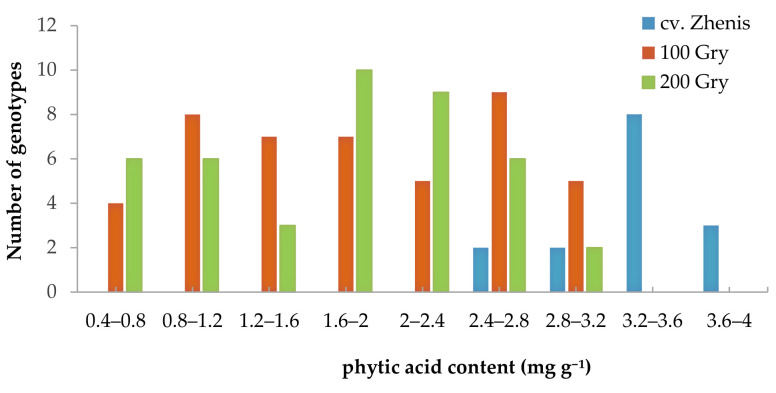
Distribution of phytic acid content (mg g^−1^) in grains of cv. Zhenis and M_5_ mutant lines developed under 100 and 200 Gry gamma irradiation.

Considerable variation in phytic acid (PA) content and its associated molar ratios with iron (Fe) and zinc (Zn) was observed among mutant lines compared to the parental cultivar cv. Zhenis, indicating a strong effect of gamma irradiation on mineral bioavailability (Figure 4; Table 3).

As shown in Figure 4, the parental cultivar exhibited a distribution skewed toward higher phytic acid levels, with the majority of genotypes concentrated within the 2.8–3.6 mg g^−1^ range and extending up to 3.6–4.0 mg g^−1^. In contrast, mutant populations displayed a clear shift toward lower phytic acid content. In the 100 Gy treatment, most genotypes were distributed within the 0.8–2.4 mg g^−1^ interval, with a noticeable reduction in the frequency of high-PA classes. This shift was even more pronounced in the 200 Gy treatment, where a substantial proportion of genotypes was concentrated in the 1.6–2.4 mg g^−1^ range, along with an increased frequency of lines in the lower intervals (0.4–1.2 mg g^−1^). These results demonstrate that gamma irradiation effectively reduces phytic acid accumulation and broadens phenotypic variation toward lower PA levels.

The impact of reduced phytic acid on mineral bioavailability was further confirmed by the analysis of phytate-to-mineral molar ratios (Table 3). The parental cultivar showed relatively high ratios, with PA:Fe = 7.05 ± 1.98 (range: 4.55–9.16) and PA:Zn = 5.52 ± 1.90 (range: 3.37–11.05), indicating limited bioavailability of these micronutrients. In contrast, mutant lines exhibited substantially lower molar ratios. In the 100 Gy-derived lines, PA:Fe decreased to 2.51 ± 1.28 (range: 0.70–6.61) and PA:Zn to 3.09 ± 1.51 (range: 0.62–6.56). A further reduction was observed in the 200 Gy population, where PA:Fe reached 2.04 ± 1.57 (range: 0.31–6.78) and PA:Zn decreased to 2.44 ± 1.64 (range: 0.59–8.78).

These reductions represent a ~2.8–3.5-fold decrease in PA:Fe and a ~1.8–2.3-fold decrease in PA:Zn ratios compared to the parent, indicating a substantial improvement in mineral bioavailability. Importantly, the lower molar ratios observed in mutant lines suggest reduced chelation of Fe and Zn by phytic acid, thereby enhancing their potential bioaccessibility in human nutrition.

Furthermore, the broader distribution of molar ratios in mutant populations reflects increased genetic variability, allowing the identification of lines combining low phytic acid content with high micronutrient content. This combination is particularly critical for biofortification, as it addresses both mineral density and bioavailability simultaneously.

Overall, these findings demonstrate that induced mutagenesis not only enhances Fe and Zn accumulation (as shown in previous sections) but also significantly reduces antinutritional constraints associated with phytic acid. This dual effect highlights the potential of selected mutant lines as promising candidates for the development of nutritionally improved wheat cultivars with enhanced mineral bioavailability.

### 3.5. Correlation Analysis

Correlation analysis revealed that the strength and direction of relationships among morphometric, nutritional, and bioavailability-related traits varied substantially depending on the irradiation dose (Table 4).

In the parental cultivar (cv. Zhenis), correlations among most traits were weak or negligible. In particular, Fe and Zn content showed only a weak positive association (r = 0.17), while their relationships with grain morphology traits, including grain area (GA) and thousand grain weight (TGW), were minimal (r ≈ 0.01–0.05). Notably, phytic acid (PA) exhibited moderate positive correlations with Fe (r = 0.43) and Zn (r = 0.41), suggesting that mineral accumulation in the control genotype is not accompanied by reduced antinutrient levels.

In contrast, mutant lines derived from 100 Gy irradiation displayed altered correlation patterns, although most relationships remained weak. A moderate negative correlation was observed between PA and Zn (r = −0.40), along with a weaker negative association between PA and Fe (r = −0.19), indicating an initial shift toward reduced antinutrient–mineral coupling. However, correlations between micronutrients and morphometric traits remained largely insignificant. More pronounced and biologically meaningful relationships emerged in mutant lines developed under 200 Gry irradiation. A significant positive correlation was detected between Fe and Zn content (r = 0.24, *p* < 0.01), indicating coordinated accumulation of micronutrients under higher mutagenic pressure. Additionally, Fe concentration showed a positive association with grain area (GA) (r = 0.19, *p* < 0.05), suggesting that increased grain size may contribute to enhanced mineral accumulation. Importantly, phytic acid exhibited negative correlations with both Fe (r = −0.06) and Zn (r = −0.06) in 200 Gry lines, while a stronger negative relationship was observed between PA and TGW (r = −0.34). These findings indicate that higher irradiation doses not only promote micronutrient accumulation but also tend to reduce antinutritional constraints, thereby improving overall grain nutritional quality.

Overall, the results demonstrate that gamma irradiation, particularly at 200 Gy, enhances the integration between grain morphology and nutritional traits while simultaneously weakening the association between phytic acid and micronutrient accumulation. This shift suggests improved biofortification potential through the combined effect of increased mineral content and reduced phytate interference.

## 4. Discussion

Phytate reduction and improved mineral bioavailability.

The present study demonstrates that gamma irradiation not only enhances grain iron (Fe) and zinc (Zn) content but also significantly reduces phytic acid (PA) content and associated antinutritional constraints, thereby improving mineral bioavailability in wheat. This dual improvement is particularly important, as increased mineral concentration alone does not guarantee enhanced nutritional value unless accompanied by reduced phytate levels [1,20,21].

The observed shift in phytic acid distribution toward lower concentration classes in mutant populations (Figure 4) indicates that induced mutagenesis effectively alters metabolic pathways involved in PA biosynthesis and accumulation [7,22,23]. In the parental cultivar, phytic acid levels were predominantly concentrated in higher intervals (2.8–3.6 mg g^−1^ and above), whereas mutant lines, particularly those derived from the 200 Gry treatment, showed a clear redistribution toward lower ranges (1.6–2.4 mg g^−1^ and even 0.4–1.2 mg g^−1^). This shift suggests that irradiation-induced mutations may disrupt key enzymatic steps in the inositol phosphate pathway, thereby limiting PA synthesis and accumulation in the grain [24,25,26].

More importantly, the reduction in phytic acid was directly reflected in significantly lower phytate-to-mineral molar ratios (Table 3), which are widely recognized as reliable indicators of mineral bioavailability [27,28,29]. The parental cultivar exhibited relatively high PA:Fe and PA:Zn ratios (7.05 and 5.52, respectively), values that are generally associated with poor mineral absorption in human nutrition. In contrast, mutant lines showed substantial reductions in these ratios, reaching mean values as low as 2.04 for PA:Fe and 2.44 for PA:Zn in the 200 Gry population. Such reductions are critical, as lower molar ratios indicate decreased chelation of Fe and Zn by phytic acid, thereby increasing their bioaccessibility during digestion [30,31,32].

Importantly, correlation analysis further supports this relationship, revealing negative associations between phytic acid and micronutrient traits (Table 4). A moderate negative correlation between PA and Zn (r = −0.40) was observed in 100 Gy-derived lines, while a negative relationship between PA and thousand grain weight (TGW) (r = −0.34) was detected in 200 Gry mutant populations. Similar negative associations between phytate and mineral accumulation have been reported in wheat and other cereals, highlighting the inhibitory role of PA in micronutrient bioavailability [33,34]. These findings indicate that reduced phytate levels are associated with both improved micronutrient accumulation and enhanced grain development under higher irradiation doses.

The magnitude of reduction observed in this study (up to ~3.5-fold for PA:Fe and ~2-fold for PA:Zn) highlights the effectiveness of induced mutagenesis as a strategy for improving not only mineral density but also its nutritional functionality. Notably, the broader range of molar ratios observed in mutant populations suggests increased genetic variability, which is essential for the selection of elite lines combining low phytate content with high micronutrient content [35].

These findings are consistent with previous reports indicating that genetic or mutagenic approaches can reduce phytic acid content in cereals and thereby enhance mineral bioavailability [36,37]. However, a key advantage of the present study lies in the simultaneous improvement of multiple traits—namely increased Fe and Zn content (Figure 2, Figure 3 and Figure 4) and reduced PA levels—without evidence of adverse trade-offs. This integrated improvement is rarely achieved in conventional breeding programs, where reductions in phytic acid are often accompanied by negative effects on seed development or yield [38,39,40].

Furthermore, the stronger coordination between Fe and Zn accumulation observed in mutant lines, particularly under the 200 Gry treatment, together with reduced phytate levels, suggests that induced mutagenesis may influence interconnected physiological pathways governing mineral uptake, transport, and storage [41]. This coordinated regulation is particularly advantageous for biofortification, as it enables the simultaneous enhancement of multiple micronutrients.

From a practical perspective, the identification of mutant lines exhibiting both elevated mineral content and reduced phytate levels represents a significant step toward the development of nutritionally improved wheat cultivars. Such genotypes are expected to deliver higher bioavailable Fe and Zn to human diets, thereby contributing to the mitigation of micronutrient deficiencies [42,43].

Overall, the results demonstrate that gamma irradiation, especially at higher doses, is an effective tool for generating wheat germplasm with improved nutritional quality, characterized by enhanced micronutrient accumulation and reduced antinutritional factors. This approach provides a valuable complementary strategy to conventional breeding and molecular techniques aimed at crop biofortification [44,45].

## 5. Conclusions

The results demonstrated that gamma irradiation effectively improved nutritional quality traits in wheat. The 100 Gry treatment was more effective for increasing grain protein content, whereas the 200 Gry treatment induced greater variability and higher accumulation of Fe and Zn. In addition, the 200 Gry dose resulted in lower phytic acid content and reduced phytate-to-Fe and phytate-to-Zn molar ratios, indicating improved mineral bioavailability.

This study demonstrates that gamma irradiation is an effective approach for improving both the nutritional quality and bioavailability of wheat grain. Significant increases in grain iron (Fe) and zinc (Zn) content were observed in mutant lines, accompanied by substantial reductions in phytic acid content and phytate-to-mineral molar ratios. In particular, Fe and Zn content showed wide variation among mutant populations, while PA:Fe and PA:Zn ratios were markedly reduced compared to the parental cultivar, indicating enhanced mineral bioavailability.

Importantly, mutant populations exhibited increased phenotypic variability, especially under the 200 Gy treatment, enabling the identification of superior genotypes combining high micronutrient content with reduced antinutritional factors. The positive association between Fe and Zn content further suggests coordinated regulation of micronutrient accumulation, while the reduction in phytate content contributes to improved nutritional functionality.

Overall, the findings highlight the potential of induced mutagenesis as a practical and efficient strategy for developing nutritionally improved wheat cultivars without compromising key agronomic traits. The mutant lines 5(4), 24(1), and 26(9) exhibited the highest grain protein content, whereas lines 45(1), 49(6), and 51(8) exhibited superior Fe and Zn accumulation. Furthermore, lines 5(4), 45(1), and 51(8) exhibited favorable agronomic traits. Mutant populations derived from both 100 Gry and 200 Gry irradiation treatments showed reduced PA:Fe and PA:Zn molar ratios, indicating improved mineral bioavailability. These lines represent valuable genetic resources for wheat biofortification and breeding programs aimed at improving grain nutritional quality, mineral bioavailability, and agronomic performance.

## Figures and Tables

**Table 1:** Yield-related traits of promising advanced mutant lines derived from 100 Gry and 200 Gry irradiation treatments in the M_5_ generation and the parent cultivar.

Genotypes	GNS	GWS (g)	GWP (g)	TGW (g)
cv. Zhenis	40.64 ± 9.27	1.57 ± 0.44	2.25 ± 0.81	38.08 ± 3.67
5(4)	43.60 ± 5.54	1.75 ± 0.25	6.91 ± 3.05 ***	49.86 ± 1.11 ***
6(4)	44.43 ± 7.29	1.70 ± 0.38	8.87 ± 4.14 ***	37.17 ± 1.75
6(5)	48.73 ± 7.35	1.99 ± 0.30	5.10 ± 1.37	33.21 ± 2.49
6(13)	46.27 ± 7.09	1.90 ± 0.32	5.75 ± 1.07	36.01 ± 2.81
13(3)	38.06 ± 11.09	1.71 ± 0.40	2.59 ± 0.83	41.02 ± 1.36
18(5)	34.02 ± 8.19	1.36 ± 0.57	7.39 ± 3.90	39.69 ± 1.85
24(1)	39.52 ± 12.20	1.13 ± 0.33	1.64 ± 0.53	38.93 ± 1.14
24(2)	43.86 ± 12.94	1.32 ± 0.30	2.64 ± 0.86	51.69 ± 1.58 ***
25(2)	45.00 ± 6.71	2.02 ± 0.37	7.23 ± 1.30	40.37 ± 1.01
26(6)	44.94 ± 7.74	2.08 ± 0.36	4.93 ± 1.99	42.51 ± 3.14
26(7)	47.38 ± 10.64	2.06 ± 0.50	4.65 ± 1.05	39.57 ± 1.51
26(9)	42.60 ± 6.90	1.85 ± 0.35	3.59 ± 1.09	43.57 ± 3.04
26(10)	47.14 ± 5.64	2.04 ± 0.29	3.67 ± 1.61	42.64 ± 0.26
30(1)	27.53 ± 10.09	1.17 ± 0.45	1.87 ± 0.62	44.65 ± 2.25
36(1)	34.17 ± 9.63	1.36 ± 0.51	2.16 ± 0.72	42.14 ± 2.83
43(1)	42.17 ± 9.60	1.64 ± 0.51	2.61 ± 0.72	44.30 ± 0.26
43(3)	39.11 ± 5.34	1.87 ± 0.63	1.69 ± 0.58	41.21 ± 1.15
43(4)	45.36 ± 4.48	1.63 ± 0.32	2.01 ± 0.34	74.65 ± 1.62 ***
45(1)	37.33 ± 6.22	1.93 ± 0.25	5.01 ± 0.28 ***	39.43 ± 1.59
45(2)	35.26 ± 7.34	1.64 ± 0.62	2.46 ± 0.37	37.23 ± 1.02
45(3)	39.48 ± 7.65	1.41 ± 0.37	2.27 ± 0.52	38.54 ± 1.39
48(3)	52.57 ± 5.88	2.10 ± 0.26	5.73 ± 1.45	42.41 ± 0.46
49(2)	52.33 ± 9.93	2.25 ± 0.47	8.91 ± 2.19	44.23 ± 3.29
49(4)	54.13 ± 9.98	2.35 ± 0.50	14.74 ± 5.69	47.66 ± 2.51 *
49(6)	47.31 ± 6.12	1.0 ± 0.67	1.96 ± 0.54	49.33 ± 2.68 **
50(7)	34.23 ± 7.95	1.53 ± 0.36	2.66 ± 0.59	44.92 ± 1.99
51(1)	48.11 ± 6.23	1.93 ± 0.28	8.99 ± 1.69	39.60 ± 2.26
51(2)	40.42 ± 8.39	1.77 ± 0.37	2.69 ± 0.83	43.64 ± 2.46
51(8)	48.11 ± 6.23	1.93 ± 0.28	8.99 ± 1.69 ***	41.71 ± 2.01
53(2)	31.82 ± 6.93	1.27 ± 0.36	1.90 ± 0.63	34.92 ± 4.73

Asterisks indicate significance at * *p* <0.05, ** *p* < 0.01 and *** *p* < 0.001.

**Table 2:** Grain size parameters of advanced M_5_ spring wheat mutant lines developed using 100 Gry and 200 Gry irradiation treatments and the parental cultivar Zhenis.

Genotypes	GA (mm^2^)	GL (mm)	GW (mm)	GL:GW Ratio
cv. Zhenis	17.41 ± 0.16	6.09 ± 0.27	3.13 ± 0.12	1.95 ± 2.25
5(4)	19.40 ± 0.13 ***	6.55 ± 0.14 *	3.50 ± 0.10 *	1.87 ± 1.40
6(4)	18.77 ± 0.08 **	6.86 ± 0.12 ***	3.52 ± 0.26 **	1.95 ± 0.46
6(5)	18.88 ± 0.09 **	6.86 ± 0.15 ***	3.61 ± 0.10 ***	1.90 ± 1.50
6(13)	18.70 ± 0.12 **	6.65 ± 0.09	3.22 ± 0.10	2.07 ± 0.90
13(3)	19.91 ± 0.14 ***	6.65 ± 0.09 **	3.50 ± 0.13 *	1.90 ± 0.69
18(5)	19.93 ± 0.15 ***	6.62 ± 0.17 **	3.73 ± 0.09 ***	1.77 ± 1.89
24(1)	19.05 ± 0.17 ***	6.55 ± 0.32 *	3.36 ± 0.13	1.94 ± 2.46
24(2)	19.14 ± 0.18 ***	6.78 ± 0.13 ***	3.85 ± 0.12 ***	1.76 ± 1.08
25(2)	19.93 ± 0.14 ***	6.82 ± 0.14 ***	3.40 ± 0.06	2.01 ± 2.33
26(6)	22.87 ± 0.15 ***	7.15 ± 0.26 ***	3.70 ± 0.13 ***	1.93 ± 2.00
26(7)	21.09 ± 0.12 ***	7.01 ± 0.01 ***	3.81 ± 0.12 ***	1.84 ± 0.08
26(9)	22.85 ± 0.16 ***	7.12 ± 0.11 ***	3.88 ± 0.01 ***	1.84 ± 11.00
26(10)	18.73 ± 0.19 **	6.67 ± 0.09 **	3.64 ± 0.17 **	1.84 ± 0.53
30(1)	18.85 ± 0.15 **	6.73 ± 0.11 **	3.57 ± 0.24 **	1.89 ± 0.46
36(1)	22.09 ± 0.12 ***	7.15 ± 0.07 ***	3.64 ± 0.21 ***	1.96 ± 0.33
43(1)	20.87 ± 0.18 ***	7.32 ± 0.23 ***	3.56 ± 0.12 ***	2.06 ± 1.92
43(3)	21.94 ± 0.15 ***	7.86 ± 0.07 ***	3.74 ± 0.20 ***	2.10 ± 0.35
43(4)	22.67 ± 0.18 ***	7.87 ± 0.06 ***	3.70 ± 0.10 ***	2.13 ± 0.60
45(1)	22.81 ± 0.17 ***	7.91 ± 0.06 ***	3.76 ± 0.10 ***	2.10 ± 0.60
45(2)	20.83 ± 0.13 ***	7.47 ± 0.19 **	3.68 ± 0.11 **	2.03 ± 1.73
45(3)	20.88 ± 0.15 ***	7.49 ± 0.10 **	3.66 ± 0.12 **	2.05 ± 0.83
48(3)	21.73 ± 0.12 ***	7.95 ± 0.16 ***	3.69 ± 0.23 ***	2.15 ± 0.70
49(2)	19.88 ± 0.11 **	7.59 ± 0.32 ***	3.64 ± 0.12 ***	2.09 ± 2.67
49(4)	21.84 ± 0.18 ***	7.83 ± 0.14 ***	3.70 ± 0.10 ***	2.12 ± 1.40
49(6)	22.94 ± 0.15 ***	7.87 ± 0.15 ***	3.74 ± 0.09 ***	2.10 ± 1.67
50(7)	22.90 ± 0.17 ***	7.91 ± 0.11 ***	3.78 ± 0.11 ***	2.09 ± 1.00
51(1)	21.84 ± 0.15 ***	8.01 ± 0.11 ***	3.86 ± 0.0 6 ***	2.08 ± 1.83
51(2)	21.96 ± 0.14 ***	8.22 ± 0.08 ***	3.80 ± 0.10 ***	2.16 ± 0.80
51(8)	22.87 ± 0.16 ***	8.09 ± 0.09	3.88 ± 0.09 ***	2.09 ± 1.00
53(2)	20.76 ± 0.16 ***	7.07 ± 0.41	3.85 ± 0.13 ***	2.08 ± 1.00 ***

Asterisks indicate significance at * *p* < 0.05, ** *p* < 0.01 and *** *p* <0.001.

**Table 3:** Phytic acid (PA) to Fe and Zn molar ratios in grains of cv. Zhenis (parent) and M_5_ mutant lines developed under 100 and 200 Gry gamma irradiation. Values are presented as mean ± SD and range.

Genotypes	ФҚ:Fe Ratio		ФҚ:Zn Ratio	
	Mean ± SD	Range	Mean ± SD	Range
cv. Zhenis (parent)	7.05 ± 1.89	4.55–9.16	5.52 ± 1.90	5.37–11.05
M5 lines (100 Gry)	2.51 ± 1.28	0.70–6.61	3.09 ± 1.51	0.62–6.56
M5 lines (200 Gry)	2.04 ± 1.57	0.31–6.78	2.44 ± 1.64	0.59–8.78

**Table 4:** Overall correlation among key traits.

	Trait	GPC	Fe	Zn	GA	TGW	PA
cv. Zhenis	Fe	0.01	1	0.17	0.01	0.01	0.43
	Zn	0.01	0.17	1	0.05	0.01	0.41
	GA	0.11	0.01	0.05	1	0.02	−0.19
	TGW	0.03	0.01	0.01	0.02	1	0.02
	PA	0.27	0.43	0.41	−0.19	0.02	1
100 Gry M_5_ lines	Fe	0.01	1	0.12	0.01	0.01	−0.19
	Zn	0.14	0.20 ***	1	0.02	0.02	−0.40
	GA	0.14	0.01	0.02	1	0.02	0.24
	TGW	0.04	0.02	0.01	0.02	1	0.45
	PA	0.17	−0.19	−0.40	0.24	0.45	1
200 Gry M_5_ lines	Fe	0.01	1	0.24 ***	0.19 *	0.01	−0.02
	Zn	0.14	0.24 ***	1	0.11 *	0.02	−0.06
	GA	0.25 *	0.19 *	0.11	1	0.05	0.15
	TGW	0.12 *	0.01	0.02	0.05	1	−0.34
	PA	0.30	−0.06	−0.06	0.15	−0.34	1

* *p* < 0.05, *** *p* < 0.001. GPC—grain protein content, Fe—iron content; Zn—zinc content; GA—grain area; TGW—thousand grain weight; PA—phytic acid.

## Data Availability

The datasets generated and analyzed during the current study are available from the corresponding author upon reasonable request.
